# Role of Transportin-SR2 in HIV-1 Nuclear Import

**DOI:** 10.3390/v13050829

**Published:** 2021-05-04

**Authors:** Maryam Tabasi, Ivan Nombela, Julie Janssens, Adrien P. Lahousse, Frauke Christ, Zeger Debyser

**Affiliations:** Laboratory for Molecular Virology and Gene Therapy, Department of Pharmaceutical and Pharmacological Sciences, KU Leuven, 3000 Leuven, Flanders, Belgium; maryam.tabasi@kuleuven.be (M.T.); ivan.nombela@kuleuven.be (I.N.); julie.janssens@kuleuven.be (J.J.); adrien.lahousse@student.kuleuven.be (A.P.L.); frauke.christ@kuleuven.be (F.C.)

**Keywords:** HIV-1, nuclear import, integrase, capsid, transportin-SR2, TRN-SR2, TNPO3, CPSF6

## Abstract

The HIV replication cycle depends on the interaction of viral proteins with proteins of the host. Unraveling host–pathogen interactions during the infection is of great importance for understanding the pathogenesis and the development of antiviral therapies. To date HIV uncoating and nuclear import are the most debated steps of the HIV-1 replication cycle. Despite numerous studies during past decades, there is still much controversy with respect to the identity and the role of viral and host factors involved in these processes. In this review, we provide a comprehensive overview on the role of transportin-SR2 as a host cell factor during active nuclear transport.

## 1. Introduction

For productive infection, most DNA viruses and a few RNA viruses including *Orthomyxoviridae* and *Retroviridae* have to pass the barrier of the nuclear membrane to deliver their genome into the nucleus. Therefore, these viruses have evolved to exploit the complex machinery of nuclear trafficking [1,2]. *Lentiviridae* such as the human immunodeficiency virus type 1 (HIV-1) are able to infect non-dividing cells like resting lymphocytes, macrophages and dendritic cells [1,3]. Classical studies showed that the nuclear envelope (NE) restricts access to the nucleus as only molecules smaller than 40 kDa or a diameter up to 5 nm can passively diffuse through the NPC [4,5]. Interestingly a recent study showed that the nuclear pore complex (NPC) represents a soft barrier to passive diffusion rather than a rigid barrier. However, the NPC contains FG domains with high net charge and low hydropathy near the cytoplasmic end of the central channel that limit the passive diffusion of macromolecules [6]. HIV-1 and other lentiviruses interact with the nuclear pores and its associated receptors and proteins through an active nuclear import mechanism that remains poorly understood. Among all HIV-1 preintegration complex (PIC) components, the viral cDNA, integrase (IN), reverse transcriptase (RT), capsid (CA), matrix antigen (MA) and viral protein R (Vpr) have all been proposed as the most important factor for HIV nuclear import [7,8,9,10,11,12]. Yet, the exact role of the viral determinants and host factors remains a subject of debate. Here we summarize the most relevant and recent studies regarding the role of the host factor transportin-SR2 (TRN-SR2 also known as transportin-3 or TNPO3) in the HIV-1 nuclear import.

## 2. The Mechanism of a Nuclear Import

**Figure 1:**
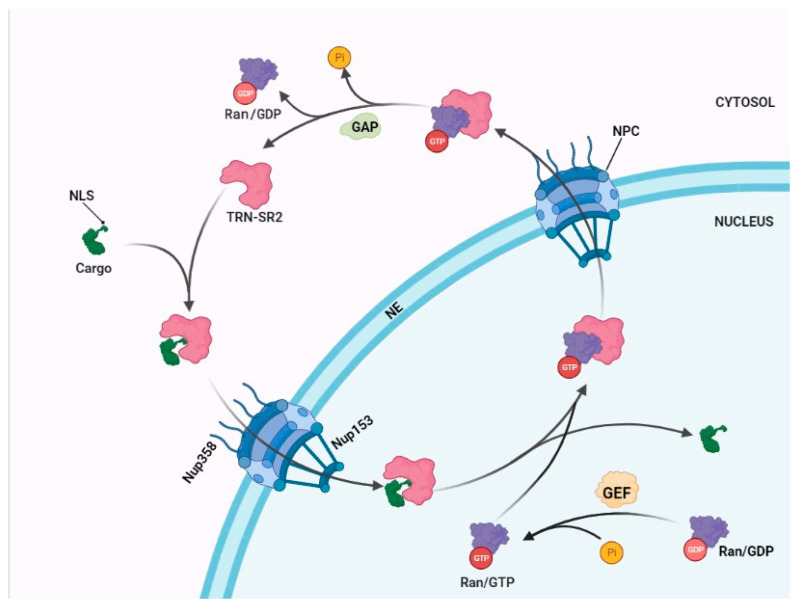
The nuclear transport cycle. In the cytoplasm, cargo/importin complex formation is mediated by the nuclear localization signal (NLS) of the cargo (upper left). In the nucleus the cargo is released upon binding of RanGTP to the importin (lower panel). Next, the importin/RanGTP complex is exported to the cytoplasm where the GTPase activating protein (GAP) hydrolyses GTP to GDP, which subsequently leads to release of importin (upper right). Ran guanine nucleotide exchange factor (GEF) phosphorylates Ran/GDP in the nucleus. The figure is created by https://app.biorender.com (accessed on 22 March 2021).

The nucleus is surrounded by the NE, a double lipid bilayer, which ensures a tight regulation of nuclear access and protection of the genetic material. Nucleocytoplasmic transport of macromolecules occurs through the NPC, which can be found with a density of 3000–5000 NPCs/nucleus on the NE of a proliferating human cell [4,13]. The NPC and the karyopherins or nuclear transport receptors are key players in the selective nuclear transport of many molecules. They are essential in the nuclear import of molecules with a size exceeding 40 kDa. Each NPC consists of almost 1000 molecules of 30 different nucleoporins (NUPs), which are conserved throughout eukaryotes. NUPs are located in the different parts of the NPC including the cytoplasmic filaments, the symmetric core, and the nuclear basket (Figure 1). They can be divided into three groups: (1) structural NUPs, (2) transmembrane NUPs (referred as Poms), and (3) FG-NUPs that contain extensive repeats of phenylalanine-glycine (FG). The FG nucleoporins such as Nup153 fill the central channel of the NPC and form a highly dynamic barrier, which determines both the selectivity and the directionality of nuclear transport. In addition, the FG repeats act as transient docking sites for importins and exportins [4,14]. Nup358/RanBP2, which has been mapped exclusively to the long cytoplasmic filaments of NPC, and Nup153, which is part of the nuclear basket and associated with chromatin, are the two most important NUPs that have been associated with HIV-1 nuclear entry [15,16,17,18,19].

Nuclear import is a tightly orchestrated process. The first step in a nuclear import is the recognition and binding of the cargo to the importin in the cytosol. Most importins belong to the β-karyopherins that interact with the cargo’s nuclear localization signal (NLS) to initiate its transport into the nucleus [4]. The Ran GTPase cycle regulates nuclear import and contributes directionality. Ran binds to GTP in the nucleus or GDP in the cytosol (Figure 1). The driving force for the cellular distribution is the concentration of Ran guanine nucleotide exchange factors (GEF) in the nucleus and GTPase-activating proteins (GAP) in the cytosol enabling directional transfer of NLS-containing cargos into the nucleus. In the next step of nuclear import, the importin-cargo complex docks to the NPC through the interaction with NUPs and passes the nuclear envelop. Inside the nucleus the binding of Ran-GTP disassembles the importin-cargo complex and releases the cargo in the cell nucleus. On the way back to the cytosol, Ran-GTP associated with importin-β is hydrolyzed to Ran-GDP to make the importin available for a new cycle of nuclear import [4,20].

## 3. Transportin-SR2 Mediates Nuclear Import

Nucleocytoplasmic transport is typically mediated by proteins of the karyopherinβ family [4]. These proteins share a similar structure consisting of an N-terminal Ran binding domain, a central NUP binding domain and a C-terminal cargo binding domain. Karyopherins recognize their cargo by a NLS or in the case of exportins by a nuclear export signal (NES). Due to an excessive amount of cargos in the cell each karyopherin can bind and carry different cargoes. Transportin-SR2 (TRN-SR2, TNPO3 or transportin-3) encoded by the *tnpo3* gene belongs to this family and has been implicated in HIV-1 nuclear import [21,22,23,24]. TRN-SR2 is a nuclear import and export protein (importin and exportin, respectively), which by binding to the serine/arginine-rich (SR) motifs in splicing factors or to an RNA recognition motif (RRM), mediates their import across the nuclear membrane. TRN-SR2 shuttles its cargoes through a Ran/GTP dependent system and its toroid structure allows interaction with a variety of cargoes [25,26].

In 2008, Brass et al. detected more than 250 HIV-1 dependency factors using a large-scale siRNA screen [22]. HIV-1 integration and infectivity were profoundly decreased with siRNAs targeting different regions of the TRN-SR2 mRNAs. At the same time Christ et al. reported the identification of TRN-SR2 as an interaction partner of HIV-1 integrase (IN) by a yeast-two-hybrid screen and showed that depletion of TRN-SR2 reduces nuclear import and replication [7,27].

## 4. Interplay between HIV-1 Integrase (IN) and TRN-SR2

HIV-1 integrase is the enzyme responsible for insertion of viral cDNA into the host genome [28]. It belongs to a family of DNA strand transferases that catalyze DNA cutting and end joining via direct transesterification. HIV-1 IN performs two enzymatic reactions: (1) it removes the 3’-GT dinucleotides from both long terminal repeats (LTRs) and (2) inserts the vDNA into the target chromatin. It is commonly accepted that at least a tetramer or even an octamer of IN is necessary to accomplish the integration step [28,29,30]. The first indication on the role of IN as a major viral determinant in HIV-1 nuclear import was presented by Gallay et al. [31]. They showed that in contrast to HIV-1 with mutations in matrix NLS and Vpr, the nuclear transport of the PICs was only profoundly inhibited when the NLS of IN was inactivated in the context of matrix NLS and Vpr defective HIV-1 variants. Later, Bouyac et al. reported on the existence of a functional NLS inside the carboxyl terminus of the catalytic core domain but outside of the catalytic triad of aspartic acid and glutamic acid residues [32]. They claimed that the IN NLS is required for infection of dividing and non-dividing cells. Mutagenesis experiments in the same study showed that viruses with a non- functional NLS were defective for nuclear import. This study confirmed that the NLS-deficient V165A and R166A IN proteins are catalytically active and thus the significant reduction in both integrated and episomal forms of viruses was attributed to a role in nuclear import [32]. However, much later it was shown that V165A and R166A are located at the interface between IN and the host factor lens epithelium-derived growth factor (LEDGF/p75), questioning their role in nuclear import [33,34,35].

Using a yeast two-hybrid screen Christ et al. were first to show that TRN-SR2 directly interacts with HIV-1 IN [7]. In their hands TRN-SR2 knockdown (KD) significantly reduced HIV-1 infection at the level of nuclear import without effect on murine leukemia virus (MLV) replication. MLV unlike HIV-1 does not have the ability to infect resting cells, but ultimately mitosis permits access to the nucleus. Christ et al. [7] and Borrenberghs et al. [36] also confirmed the role of TRN-SR2 in HIV-1 nuclear import by using fluorescently labeled viruses. Microscopy analysis revealed a clear reduction in nuclear PICs in TRN-SR2 KD cells confirming the role of this factor in HIV-1 nuclear entry. Taltynov et al. by using size exclusion chromatography showed that recombinant HIV-1 IN was displaced from TRN-SR2 upon binding of RanGTP to the complex implying the direct interaction between TRN-SR2 and IN [37].

In an attempt to map the interface between HIV-1 integrase and TRN-SR2 De Houwer et al. introduced a double mutation in IN (IN^-R263A/K264A^) and studied its effect on HIV-1 nuclear import by confocal microscopy of fluorescently labeled viral particles [38]. The IN^-R263A/K264A^ mutant virus was impaired in HIV-1 replication at the level of nuclear import. Hultquist et al. used clustered regularly interspaced short palindromic repeats/Cas9 (CRISPR/Cas9) to deplete either HIV-1 coreceptors (CXCR4 and CCR5) or dependency factors including TRN-SR2, and LEDGF in primary human T cells [39]. Results showed that CXCR4 and CCR5 knockout (KO) cell were resistant to HIV-1 infection in a tropism-dependent manner whereas TRN-SR2 and LEDGF KO cells led to a reduced infection independent of tropism confirming the vital role of these cofactors in HIV-1 infection. It was shown that type l interferon-inducible microRNA (miR), miR-128, directly targets TRN-SR2 mRNA and downregulates the protein expression [40]. Overexpression or downregulation of miR-128 in cell lines and primary CD4+ T cells inhibited WT HIV-1 infection but not MLV infections. The N74D HIV-1 mutant was also repressed by overexpression of miR-128 but less than WT HIV-1.

Interestingly, AlphaScreen-based high-throughput screening revealed specific small molecule inhibitors of the IN/TRN-SR2 interaction [41]. At a concentration of 100 µM these compounds significantly reduced the number of nuclear PICs compared to both DMSO and raltegravir controls. These results support the role of the IN/TRN-SR2 interaction in nuclear import.

Apart from HIV-1 other retroviruses show TRN-SR2 dependency. Faysal Bin et al. for example studied the role of TRN-SR2 in the prototype foamy virus (PFV) life cycle [42]. TRN-SR2 KD cells significantly reduced the infection of PFV (15–20%) compared to the WT cells. The authors showed that the numbers of both 1-LTR and 2-LTR circles were slightly increased in TRN-SR2 KD cells pointing to the possible role of TRN-SR2 in nuclear import or integration steps of PFV.

Despite various studies pointing toward an important role of HIV-1 IN [7,36,38,43,44,45,46] in the nuclear translocation of PICs, questions with respect to the direct interaction between IN and TRN-SR2 remain. Indeed, IN does not contain a serine–arginine-rich motif and therefore is not a classical cargo of TRN-SR2. However, it has been shown that TRN-SR2 also imports proteins without SR such as stem–loop binding protein (SLBP) [47]. It was shown that the RNA recognition motif (RRM) also known as the RNA-binding domain (RBD) is sufficient for binding of RNA molecules to the protein [48]. One earlier study has shown that subnuclear localization of polypyrimidine tract binding protein-associated splicing factor (PSF) depends on the RRM [49]. TRN-SR2 is able to bind a wide range of RRM containing proteins and crystallography has revealed that TRN-SR2 interacts with both the RRM and the arginine-serine domain of alternative splicing factor (ASF) pre-mRNA-splicing factor SF2 (ASF/SF2) and cleavage and polyadenylation specificity factor 6 (CPSF6) [26].

Several models explaining the interaction between HIV-1 IN and TRN-SR2 have been proposed. First, various independent groups confirmed the direct interaction between TRN-SR2 and HIV-1 IN using different approaches such as AlphaScreen^®^ and immunoprecipitation [7,8,44,45,50]. De Houwer et al. proposed that the catalytic core domain (CCD) and the C-terminal domain (CTD) of IN are implicated in this interaction [44]. Later, Tsirkone et al. hypothesized that TRN-SR2 interacts in a non-canonical way by forming a triple complex including IN/TRN-SR2 and natural cargoes of TRN-SR2 [51]. Size-exclusion chromatography was used to show that TRN-SR2 forms a stable complex with the CCD-CTD construct of HIV-1 integrase at a 1:2 molar ratio. The same study also reported that IN interacts with the N-terminal half of TRN-SR2 through the HEAT repeats 4, 10, and 11. The same study reported on a small-angle X-ray scattering (SAXS) based 3D model of the TRN-SR2/CCD-CTD complex. The SAXS data showed that the TRN-SR2/CCD-CTD complex is considerably less compact than the TRN-SR2/ASF/SF2 or TRN-SR2/RanGTP complexes suggesting a more open solenoid structure of TRN-SR2. The authors also demonstrated that the IN/TRN-SR2 interaction does not involve the SR-binding region, and thus proposed that IN can piggyback during the TRN-SR2-mediated nuclear import of cellular cargoes, such as ASF/SF2 and CPSF6. Interestingly, Rice et al. found that TRN-SR2 binds directly to Gag protein of avian alpharetrovirus rous sarcoma virus (RSV) while the cargo-binding domain of TRN-SR2 was not required in this interaction implying that RSV indeed hijacks TRN-SR2 while allowing simultaneous binding of natural host cargos to it [52]. 

Some studies reported that the IN/TRN-SR2 interaction is important in other stages of infection rather than nuclear import. Zhou et al. for example showed that TRN-SR2 is required for HIV-1 integration [53]. Alu-qPCR data showed a 10-fold reduction in integrated HIV-1 DNA in TRN-SR2 KD cells. Similarly, Cribier et al. used IN mutants W131A and Q168L, known to be impaired for interaction with both TRN-SR2 and LEDGF/p75, to study the impact of these mutations on PIC nuclear import. Quantification of 2-LTR circles with these IN mutants showed a rather limited effect (W131A) or no effect (Q168L). This phenotype may be masked by the effect of the mutations on the interaction with LEDGF/p75, which affects integration [50]. However, these mutants are known to be defective in the interaction with LEDGF/p75 [33,54] questioning the interpretation of the data. Interestingly, Melia et al. identified that a heterozygous single nucleotide deletion (c.2771del) in the stop codon of TRN-SR2 is the cause of limb girdle muscular dystrophy 1F (LGMD1F) [55]. Recently a similar study found that a 15-amino acid extension of the C-terminal of TRN-SR2 causing LGMD1F significantly reduced HIV-1 infection and integration. However, analysis of 2-LTR episomes did not reveal a difference between patient and control groups, indicating that other steps than nuclear import may be affected [56]. 

## 5. Interaction between HIV-1 Capsid and TRN-SR2

HIV-1 capsid (CA) plays a vital role in the life cycle and infection of the virus. One of its primary roles is to protect the viral ribonucleoprotein (RNP) and eventually promote its transfer to the cell nucleus for integration into the host genome [57]. The CA protein is derived from viral Gag and Gag-Pol polyproteins the peptide bonds of which are cleaved by the viral protease during maturation leading to a cone shaped structure composed of 250 CA hexamers and 12 pentamers [58]. Several studies have questioned the role of HIV IN in nuclear import and suggested that viral CA is the critical determinant for entering the cell nucleus [8,59]. An earlier study reported that chimeric HIV-1 carrying MLV encoding sequences for MA, p12 and CA failed to efficiently infect nondividing cells. Since a significant reduction of nuclear episomes was observed, the authors concluded that CA is the major determinant of HIV-1 nuclear import [60]. The authors later showed that virus harboring the Q63A/Q67A mutation in the N-terminal domain of CA lost the ability to infect nondividing cells including primary macrophages [61].

Employing these chimeric MLV/HIV-1 viruses, it was suggested that HIV-1 CA is the dominant factor for TRN-SR2 dependency during the HIV-1 nuclear import [8]. The authors confirmed that retroviral infectivity depends on cellular TRN-SR2 but claimed that insensitivity of chimeric viruses to TRN-SR2 depletion maps to CA as resensitization occurs after exchanging the MLV CA protein for that of HIV-1. In the same study it was demonstrated that MLV integrase associates with TRN-SR2 in pull down assays but depletion of TRN-SR2 did not affect MLV infectivity [8]. This result is in line with the dependence of MLV infection on mitosis and therefore disintegration of the nuclear envelope. With respect to the MLV IN/TRN-SR2 interaction conflicting results have been presented. Why TRN-SR2 interacts with MLV integrase in vitro but plays no role in MLV infection is not understood. For some authors it questions the relevance of the direct interaction between HIV IN and TRN-SR2 [8]. Generally, the results with chimeric viruses have to be interpreted carefully as the replication of these chimers is poor. Moreover, the CA-mediated effects on virus uncoating may indirectly affect the interaction between IN and TRN-SR2 since IN is located within the capsid cone.

For a better understanding of the role of the CA/TRN-SR2 interaction in HIV-1 nuclear import, 27 single-cycle HIV-1 vectors each carrying a different CA mutation were engineered [59]. Infection of TRN-SR2 KD cells with HIV-1 vectors was 11-fold reduced compared to WT control cell lines. Fourteen CA mutants were relatively TRN-SR2-independent, as compared to WT HIV-1. Two mutants were more TRN-SR2-dependent than the WT, and eleven mutants were actually inhibited by TRN-SR2. In that paper it was originally reported that TRN-SR2 depletion does not have any significant effect on the amount of 2-LTR circles produced during HIV-1 infection. Yet, in a follow up paper the same authors admitted the complexity of 2-LTR detection questioning their previous results [62]. 2-LTR detection by qPCR is technically challenging. In fact, a control with raltegravir, known to increase 2-LTR circles, should be included. Only if no increase in 2-LTR circles is observed in the presence of raltegravir a defect in the nuclear import can be claimed. Based on their results with CA mutants, the authors concluded that CA is the main determinant of TRN-SR2 sensitivity and they proposed a role in the nucleus to promote the viral life cycle [59]. 

Yet, so far, no strong evidence for a direct interaction between TRN-SR2 and CA has been reported. Larue et al. studied the interaction of TRN-SR2 with IN and CA [45]. In this study biophysical assays including circular dichroism, analytical ultracentrifugation, SAXS and homology modeling were used to characterize the TRN-SR2 structure. In vitro biochemical binding techniques confirmed the interaction of IN and TRN-SR2, while no direct interaction between TRN-SR2 and CA was detected.

Similar to the debate about the IN/TRN-SR2 interaction, it was proposed that CA/TRN-SR2 interaction can be indirect and/or affect other replication steps than nuclear import. Along this line Valle-Casuso et al. found that TRN-SR2 binds to in vitro-assembled HIV-1 capsid-nucleocapsid (CA-NC) complexes and this interaction is required for the successful HIV-1 integration into the host genome [63]. Alu-PCR data indicated a 20-fold decrease of integration in TRN-SR2 KD cells. The authors also showed that HIV-1 infection in TRN-SR2 KD and control cells displayed similar levels of 2-LTR circles. Therefore, based on their results it was suggested that interaction of HIV-1 CA and TRN-SR2 leads to the proper maturation of the PIC that later results in successful integration of viral DNA into the host chromosome. Ocwieja et al. suggested that HIV-1 CA interaction with TRN-SR2 and NUP358 influences viral integration targeting as depletion of these cellular factors reduced HIV integration frequency in gene-dense regions and near gene-associated features [64]. 

**Figure 2:**
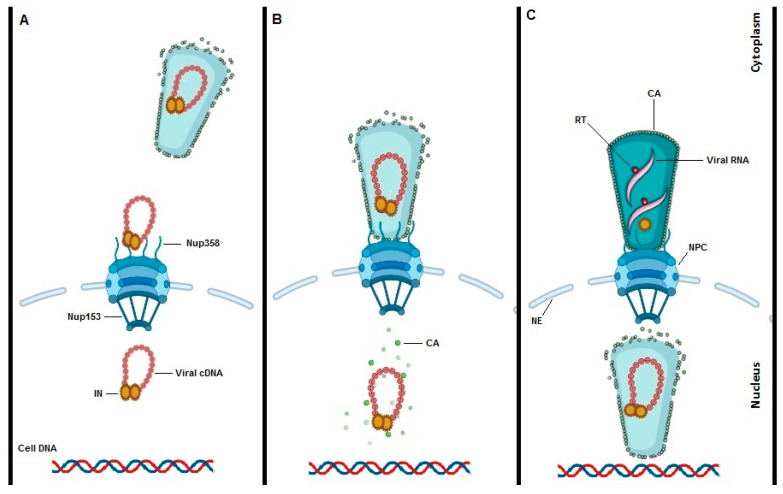
Models of HIV-1 uncoating. To date three potential models for HIV-1 uncoating are proposed. “Immediate uncoating” points to the early disassembly of the HIV-1 core inside the cytoplasm during the first hour of infection and prior to NPC docking (**A**). Uncoating at the NE is supported by imaging experiments showing that uncoating occurs when the core is stalled at the NPC. (**B**). Recent studies are claiming a “nuclear uncoating” mechanism and represent the third model of HIV-1 uncoating (**C**). In the third model, intact HIV-1 cones enter into the cell nucleus, reverse transcription is completed, and uncoating and integration are all completed inside the nucleus and close to the integration site.

Since the size of the conical HIV-1 core (119 ± 11 nm in length and 60 ± 8 nm in width) [65] exceeds the generally accepted NPC central channel diameter of 40 nm [66], it is believed that viral cone integrity is lost prior to nuclear import. Several labs reported that intranuclear PICs are CA-positive in different cell types including primary human macrophages [67,68,69]. Hulme et al. used superresolution structural illumination microscopy (SIM) to study the intensity of CA signal associated with IN-GFP in nuclear viral particles [67]. They found that only a subset of CA proteins remains associated with PICs after nuclear import indicating that intact capsids cannot enter the nucleus. However, this study could not determine where exactly uncoating occurs nor whether it is a slow progressive or rapid process. The authors in the follow up studies proposed a model of uncoating in which disassociation of CA from viral particles is initiated by reverse transcription in the cytoplasm and within the first hour of infection (Figure 2A) [70,71].

Later, Francis et al. showed by using live-cell imaging and single particle tracking that CA mediates docking of PICs to the NE and only particles that lost CypA-DsRed/CA (referred to as uncoating) during the docking period were able to enter the nucleus and to establish infection [69]. This study also reported that the N74D CA mutant, which no longer binds to CPSF6 and NUP153, slowly uncoats during NE residency; yet the nuclear penetration of the mutant is severely limited. Using viral particles labeled with CA-eGFP, Zurnic et al. found that 18–23% of all cytosolic IN-mCherry complexes contain CA-eGFP, which slightly decreased to 15% when reaching the NE [68]. However, only 3% of CA-eGFP complexes in the nucleus contained IN-mCherry implying that the dissociation of CA-eGFP from IN-mCherry complexes (uncoating) occurs at the NE (Figure 2B).

Recent studies claim that HIV-1 particles with an intact or remodeled capsid core are able to enter the nucleus (Figure 2C). Confocal live cell imaging by Burdick et al. revealed that these intact viral cores while interacting with CPSF6 enter the cell nucleus [72]. However, the results of Zurnic et al. [68] and Francis et al. [69] are at odds with these findings since they consistently see a reduction of CA proteins inside the nucleus. Of note, the first clear data on the presence of multiple CA proteins complexes reaching the nucleus of HeLa cells and more importantly of primary lymphocytes have been published by Blanco-Rodriguez et al. [73]. This study shows a remodeling of the viral core during nuclear translocation. Later Zila et al. studied HIV-1 core structures during nuclear import in CD4+ cell lines by using correlative light electron microscopy (CLEM) and cryo-electron tomography (cryo-ET) [74]. They claim that 85% of HIV-1 particles retain their cone shaped capsid during NE residency and even pass through the NPC, but that the overall shape is lost inside the nucleus. They also reported that the NPC is enlarged in lymphocytes and has an average diameter of 64 nm. However, these EM experiments are technically limited by the number of particles measured. Whether the images are representative for functional particles capable of integration is difficult to judge. Moreover, integration-deficient virus or CPSF6-independent mutants were used to overcome the technical limitations of the experiments.

Recently, some studies provided evidence that HIV-1 reverse transcription is completed inside the nucleus [72,75,76,77,78]. Francis et al. employing inhibitors including nevirapine, raltegravir and PF74 targeting different steps of the HIV-1 replication cycle, showed that viral replicative complexes that established the infection lost capsid during residency at the nucleopore and prior to completion of viral DNA synthesis [76]. Dharan et al. by using an inducible nuclear pore complex blockade, namely Nup62 fused to a drug-inducible dimerization domain B (DmrB), found that despite the completion of reverse transcription in the nucleus, some degree of cytoplasmic reverse transcription is required for a productive infection [78]. Interestingly, Rensen et al. showed that viral RNA genomes accumulate in particular nuclear niches in macrophages under the nevirapine pressure [77]. Once the drug is released a nuclear RT is restored, indicating that the RT can start and end in infected macrophages.

## 6. Interplay between TRN-SR2 and CPSF6

CPSF6 is one subunit of a cellular complex required for maturation of messenger RNAs (mRNA) from pre-mRNA [79]. CPSF6 directly activates the mRNA 3′-processing machinery and has an important role in mRNA nuclear export as well. CPSF6 has been implicated in several steps in the HIV-1 life cycle including cytoplasmic trafficking, nuclear import and integration site selection [80,81,82].

Lee et al. found that a fragment of CPSF6 (mCPSF6-358 lacking the RS domain) prevents HIV-1 nuclear entry by targeting the viral CA [16]. The authors also showed that the N74D CA mutant could evade mCPSF6-358 blockage of nuclear import. In addition, they claimed that infection with HIV-1 N74D is independent from TRN-SR2 and NUP153. Based on these data the authors proposed that HIV-1 nuclear import is flexible and CA modulates interactions with the nuclear transport machinery. X-ray crystallography has shown that a group of charged residues on and around the arginine-rich helix of the TRN-SR2 HEAT repeat 15 occupies the phosphorylated SR domain and leads to nuclear import of splicing factors like CPSF6 [26]. Mutations in this region impair TRN-SR2 interaction with CPSF6 and block HIV-1 replication. Likewise, Logue et al. found that mutations in the carboxy terminus of the cargo binding domain in TRN-SR2 prevented HIV-1 infection [83]. Further analysis in the same study by using confocal microscopy using TRN-SR2 fused to enhanced green fluorescent protein (eGFP) confirmed that TRN-SR2/eGFP localized into the nucleus, indicating that the defect in viral infection is because of a loss in binding ability to a cellular or viral cargo.

X-ray crystallography further demonstrated that CPSF6 binds to a protein–protein interface on the N-terminal domain of intact HIV-1 CA hexamers [84]. Mutagenesis studies showed that the CA binding domains of CPSF6 also determine TRN-SR2 and NUP358 dependency. Adding a NLS motif to CPSF6-358 restores its trafficking into the nucleus and the HIV-1 infectivity. These authors suggested that the direct interaction of CA with host cofactors like CPSF6 and TRN-SR2 mediates HIV-1 nuclear import.

In another study HIV-1 WT and CA mutants were used to infect TRN-SR2 KD cells for addressing the role of TRN-SR2 in HIV-1 nuclear import [62]. In control cells CPSF6 was exclusively found inside the nucleus, but in the absence of TRN-SR2 endogenous CPSF6 was also detectable in the cytoplasm. Based on these data the conclusion was made that inhibition of HIV-1 infection by TRN-SR2 KD is caused by cytoplasmic localization of CPSF6 and subsequent hyperstabilization of the CA cone hence attributing an indirect role to TRN-SR2 in HIV-1 nuclear import. Fricke et al. showed that TRN-SR2 depletion inhibits HIV-1 infection while simultaneous depletion of CPSF6 and TRN-SR2 rescues HIV-1 infection showing that the ability of TRN-SR2 KD cells to block HIV-1 infection depends on CPSF6 [85]. They also showed that TRN-SR2 depletion mislocalizes ASF/SF2 but not CPSF6. The authors did not detect a 2-LTR increase in TRN-SR2 KD cells in comparison to raltegravir treated cells. They concluded that cytosolic CPSF6 restricts HIV-1 infection before or during nuclear import.

Although several studies claim that the HIV-1 CA mutant N74D is independent of TRN-SR2 for nuclear import and probably is using a different route than HIV-1 WT [16,59] some results are at odds with this hypothesis. Thys et al., for example, compared the TRN-SR2 dependence of the HIV-1 N74D CA mutant carrying the HIV-1 envelope with the same mutant pseudotyped with VSV-g [46]. The authors found that the CA N74D mutant dependence on TRN-SR2 is directly affected by the viral envelope used. They suggested that pseudotyping the CA N74D mutant virus with VSV-g may affect uncoating or docking to the nuclear pore that precedes the interaction between IN and TRN-SR2. Finally, this study concluded that an alternative nuclear import is being used when the N74D CA mutant is entering the cell via pH-dependent endocytosis. Moreover, Thys et al. showed that multiple round replication of N74D still strongly depended on the presence of TRN-SR2 [46]. No explanation for the discrepancy between single and multiple round replication has been given.

Price et al. found that CPSF6 and NUP153 have an overlapping binding site, which is actually part of a larger protein–protein interface that only exists in a hexameric CA [86]. Zhou et al. showed that combination of following substitutions including S41A, Q67H, and either V165I or L172I in HIV-1 CA causes strong resistance to PF74 [87]. The PF74 resistant mutant proved less dependent on TRN-SR2, CPSF6, Nup153, and Nup358. Authors proposed that PF74 resistance is the result of HIV-1 evolution in response to an alteration of the viral dependence on host cofactors involving in nuclear entry.

By two-color stimulated-emission-depletion microscopy it was shown that CPSF6 depletion in macrophages leads to the accumulation of viral particles at the nuclear pore and reduction of infectivity [81]. The authors reported that in the absence of CPSF6 these arrested viral particles reside at the nuclear pore and without release from the nuclear basket eventually integrate their DNA into the lamina-associated heterochromatin. This may explain why HIV-1 mutants such as N74D that are defective for binding to CPSF6 still are able to establish productive infection [88]. 

By using confocal microscopy and live cell imaging Burdick et al. showed that in contrast to WT particles, N74D or A77V HIV-1 mutants, which have reduced affinity for binding to CPSF6, have much longer NE residency time revealing either an uncoating or a nuclear import deficit [72]. Unlike WT particles these mutants uncoated during the docking period at the NE and subsequently inserted their genome in peripheral chromatin close to the NE. The authors suggest that recruitment of CPSF6 by PICs during NE residence results in an alteration of the NPC or the viral core structure to facilitate the nuclear import of intact viral particles. Recent studies have shown that IN strongly colocalizes with CPSF6 clusters in CD4+T lymphocytes and macrophage-like cells. During HIV infection CPSF6 relocated to the vicinity of lens epithelium-derived growth factor 75 (LEDGF/p75), which is known to be required for HIV-1 integration [89]. Another study showed that HIV-1 capsid traffics towards the nucleus with CPSF6 and its binding partner TRN-SR2 on microtubules [80]. 

One recent study found that the Sec24C, a protein involved in vesicular transport [90], interacts and cotraffics with HIV-1 IN and CA labeled complexes [91]. Depletion of Sec24C reduced RT, nuclear import and integration. The authors also showed that Sec24CΔFG effectively displaced cellular CPSF6 and Nup153 from CA tubes, whereas PF74 inhibited Sec24CΔFG binding to HIV-1 cores with a half maximal inhibitory concentration (IC_50_) of 0.692 µM. Capsid inhibitors such as PF74 affect the CA core stability in a concentration dependent manner [92,93]. PF74 stabilizes viral cores [94,95] at low concentration (≤2 µM), but exerts the opposite effect at a concentration of 10 µM [96]. Its mechanism of action remains partially unknown [86,97]. A long-acting CA inhibitor with clinical potency has recently been reported [98]. Structural studies revealed that Sec24C, CPSF6, NUP153 and PF74 share the same binding interface on CA [91]. In conclusion, both the N74D mutation and the CA inhibitors have pleiotropic effects affecting the interaction of CA with consecutively Sec24C, Nup153 and CPSF6. One should avoid using the N74D mutant virus as a surrogate for CPSF6 depletion. It is shown that nuclear HIV-1 PICs are directed to nuclear speckles a nuclear region with high expression of splicing factors [99]. Interaction between CPSF6 and CA is required for transporting HIV-1 reverse-transcription- and preintegration-complexes (RTC/PIC) to the nuclear speckles.

In summary, these studies suggest an indirect role of TRN-SR2 in the nuclear import of HIV-1 RTC/PIC as the import factor of CPSF6, and dismiss a role for IN. This model proposes that CPSF6 mediates HIV-1 nuclear import and integration site selection, based on its ability to interact with HIV-1 CA [16,84].

## 7. Cyclophilin A and HIV-1 Nuclear Import

Cyclophilins (Cyps), also known as immunophilins, are members of an ubiquitous family that are known to bind cyclosporine and catalyze *trans* to *cis* peptidyl-prolyl isomerization affecting protein folding [100]. Human cells have 16 Cyps and CypA is the first host protein, which was found to be incorporated into HIV-1 virions through the interaction with CA [59]. The CA/CypA interaction is conserved throughout the evolution of lentiviruses as the ancient endogenous lentiviruses contain CypA binding motifs [101]. Ever since many studies have tried to understand the impact of this interaction on the virus replication cycle [102,103,104,105,106].

Shah et al. found that TRN-SR2 and CypA have opposite effects on HIV-1 uncoating [107]. This study reported that TRN-SR2 stimulates HIV-1 uncoating while CypA directly inhibits HIV-1 uncoating. In this study TRN-SR2 and CypA were proposed as modulators of HIV-1 uncoating. Several studies found that CA binds directly to the CypA homology domain of Nup358/RanBP2 and they revealed that the interaction of CypA/CA results in docking to the NE and translocation into the cell nucleus [15,108]. One of the possible roles of the CypA/CA interaction might be the regulation of the CA/Nup358 interaction that can lead to nuclear envelop docking, uncoating and nuclear entry. One study concluded that CA/CypA interaction might promote the reverse transcription stage, but at the same time block nuclear import by maintaining capsid integrity and delaying uncoating [106]. A recent study has reported that disruption of HIV-1 capsid interaction with CypA results in accumulation of CPSF6 in the cytoplasm and blocking of viral infection [109]. This study suggests that CypA prevents the HIV-1 capsid from premature binding to cytoplasmic CPSF6.

## 8. Conclusions: Towards a Model for HIV-1 Nuclear Entry

It is difficult to ignore the process of HIV-1 uncoating when talking about HIV-1 nuclear import. Since, HIV-1 uncoating is key in understanding nuclear import, any nuclear import model will depend on the model of uncoating chosen. To date three different models of HIV-1 uncoating are put forward with data in favor or against (Figure 2). The first model proposes early uncoating shortly after HIV-1 fusion with the cell membrane and the release of the viral core into the cytoplasm (Figure 2A) [67,70,71,110]. However, based on live cell imaging and single particle tracking, the early uncoating hypothesis is not any longer accepted as the prevailing model. The second HIV-1 uncoating mechanism is based on studies from Francis et al. and studies from our group [36,68,69] and proposes uncoating to take place at the NE while the viral core is docked to the NPC (Figure 2B). The mechanism of action of PF74 that induces premature uncoating and inhibits the RT step was consistent with this model. Alternatively, PF74 may block the interaction of N74D in CA with Nup153 and/or CPSF6 blocking nuclear import. That mechanism would be consistent with the second or third uncoating models. This model is consistent with the reduction of IN content detected in nuclear versus cytoplasmic PICs [36,111] and the LEDGF/p75-mediated increase in FRET ratio of IN upon nuclear entry [36]. The model is also consistent with a reduction in the amount of CA associated with nuclear PICs, although staining may be confounded by interaction partners such as CPSF6. Of note capsid proteins may reassemble inside the nucleus, as is the case with the hepatitis B virus [2]. In 2020 Burdick et al. presented a third model of uncoating whereby intact cones enter the nucleus, complete reverse transcription and integration takes place 1–2 h after uncoating (Figure 2C) [72,75]. The mechanism of action of PF74 that induces premature uncoating at higher concentrations and inhibits the RT step was consistent with the second model. 

**Figure 3:**
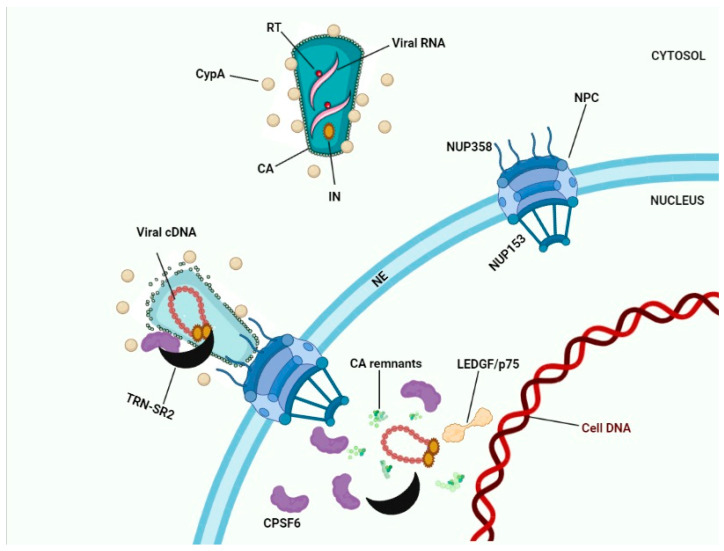
HIV-1 nuclear import. Interaction between Nup358 and viral CA results in docking of HIV-1 cones to the NE. Depending on the model chosen (see Figure 2) capsid uncoating occurs in the cytoplasm, at the NE, or in the nucleus. Interaction between Nup153 and CA results in docking to the nuclear basket of the NPC. CPSF6 bound to CA proteins may recruit TRN-SR2 to the PICs. After capsid uncoating the triple complex of transportin-SR2/HIV-1 IN/CPSF6 may subsequently translocate the viral integration complex into the nucleus. Binding of nuclear CPSF6 molecules to CA remnants may release these remnants into the nucleoplasm.

We present our favorite scenario for HIV-1 nuclear import (Figure 3) although the exact details still need to be clarified. Cytoplasmic filaments of NUPs mainly consisting of the NUP358 interacting with the CypA binding motif in HIV-1 CA and facilitating docking to the NE. During their residency at the NPC CA interacted with NUP153 in the nuclear basket. Physical stress following completion of reverse transcription shears the narrow end of the cone residing in the nuclear basket. CPSF6 may be recruited to the NPC by its physical interaction with CA. The HIV-1 IN complex interacted with the HEAT repeats in the N-terminal half of TRN-SR2 and since this interaction did not involve the SR-binding region, PICs can be smuggled during TRN-SR2-mediated nuclear import of CPSF6. Nuclear CPSF6 competed with the interaction of Nup153 with the CA residues in the cone stuck in the NPC. Competition between nuclear CPSF6 released the capsid remnants from the nuclear basket (Figure 3). Ran-GTP dissociates IN from TRN-SR2. LEDGF/p75 altered the IN conformation prior to integration. In the absence of CPSF6 (by depletion of for the N74D mutant) this nuclear import pathway was aborted; instead, PICs integrated while still associated with the basket of the NPC.
